# 3-D conformal radiotherapy with concomitant and adjuvant temozolomide for patients with glioblastoma multiforme and evaluation of prognostic factors

**DOI:** 10.2478/v10019-011-0019-2

**Published:** 2011-07-20

**Authors:** Yilmaz Tezcan, Mehmet Koc

**Affiliations:** Department of Radiation Oncology, Meram Faculty of Medicine, Selcuk University, Konya, Turkey

**Keywords:** glioblastoma multiforme, conformal radiotherapy, temozolomide, prognostic factors

## Abstract

**Background:**

The aim of the retrospective study was to evaluate the outcome and prognostic factors of newly diagnosed glioblastoma patients who received 3-D conformal radiotherapy (RT) combined with concomitant and/or adjuvant temozalamide (TMZ) postoperatively.

**Patients and methods:**

Fifty patients with glioblastoma multiforme were treated with 3-D conformal RT combined with concomitant and/or adjuvant TMZ postoperatively. Median age was 57 years (range, 12–79) and median Karnofsky performance status (KPS) was 70 (range, 40–100). A multivariate Cox regression model was used to test the effect of age, sex, KPS, extent of surgery, tumour dimension (<5cm *vs.* ≥5cm), full dose RT (≥60 Gy *vs.* <60 Gy), concurrent TMZ and adjuvant TMZ treatment (adjuvant therapy plus 6 cycles of TMZ group versus <6 cycles of TMZ group) on the overall survival.

**Results:**

The median follow up time was 10 months (range 3–42). One- and 2-year overall survival rates were 46% and 20%, respectively. The prognostic factors important for the overall survival were a full dose RT (≥60 Gy) (p=0.005) and the application of adjuvant TMZ for 6 cycles (p=0.009).

**Conclusions:**

The results of our study confirm the efficiency of RT plus concomitant and adjuvant TMZ, with an acceptable toxicity in patients. We suggest that at least 6 cycles of adjuvant TMZ should be administered to obtain a benefit from the adjuvant treatment.

## Introduction

Glioblastoma is the most common primary brain tumour in adults and accounts for approximately 60–70% of all gliomas.^1^–^3^ The incidence of malignant glioma is increasing among elderly patients.^4^ Malignant glioma may develop at all ages, it can also occur in children, but the peak incidence being in the fifth and sixth decades of life.^5^ Malignant gliomas are 40% more common in men than in women and twice as common in whites as in blacks. The median age of patients at the time of diagnosis is 64 years in the case of glioblastomas. Median survival time is less than 1 year after diagnosis.^1^,^2^

A recent meta-analysis based on 12 randomized trials showed a small survival benefit (6% in the one year survival rate, from 40% to 46%) from the addition of alkylating agents.^7^ Until recently, treatment options for patients with malignant glioma were limited and mainly the same for all subtypes of malignant glioma. The treatment included surgery to the extent feasible and radiotherapy (RT). Chemotherapy (CT) used as the adjuvant treatment or at recurrence had a marginal role.^8^–^10^

Concomitant and adjuvant temozolomide (TMZ) CT significantly improved median, 2- and 5-year survival in a large randomized trial, and is the current standard of care for patients with glioblastoma up to age 70.^3^,^11^ No randomized data are available for elderly patients (>70 years) with a good performance status. The contribution of adjuvant TMZ and the optimal cycle schedule is also still not known.

Prognostic factors of glioblastoma multiforme (GBM) and anaplastic astrocytomas include age at diagnosis, Karnofsky performance status (KPS), histology, extent of resection, duration of symptoms, and neurologic functional/mental status.^12^

In the present study, we evaluated the outcome of newly diagnosed glioblastoma patients who received 3-D conformal RT combined with concomitant and/or adjuvant TMZ postoperatively. Additionally, prognostic factors and obtaining a benefit from 6 cycles of adjuvant TMZ for the survival were evaluated.

## Patients and methods

### Patient

Between December 2005 and August 2010, 50 patients with GBM were treated with 3-D conformal RT combined with concomitant and/or adjuvant TMZ postoperatively. The median age was 57 years (range, 12–79) and the male: female ratio was 2.1:1. All of the patients had computed tomography scan or magnetic resonance imaging preoperatively. Median KPS was 70 (range, 40–100) before RT.

### Tumour characteristics

Median tumour diameter was 5 cm (range; 1–12 cm), patients with tumour dimensions less than 5 cm were 32% and ≥5 cm were 68%. The location of the tumour was temporal in 40%, parietal in 30%, frontal in 24%, occipital in 6% and others in 11%, respectively.

### Treatment

The surgery was limited to stereotactic biopsy in 28% of the patients, the subtotal resection was performed in 18%, the gross total resection in 44%, and only the radiologic diagnosis was done in 10% of the patients. The median interval between surgery and radiotherapy was 38 days (range; 19–74 days).

3-D conformal RT, delivered by linear accelerators with a 6 mV or more energy, consisted of the fractionated focal irradiation, at a dose of 2 Gy per fraction, given once daily 5 days per week. The median total RT dose was 60 Gy (range; 30–68 Gy). The dose was defined according to the guidelines of the International Commission on Radiation Units and Measurements. The initial radiotherapy field for 44–46 Gy was planned according to preoperative and postoperative cranial magnetic resonance imaging data. We used CT planning with the use of contrast media. The clinical target volume was determined as gross tumour volume plus surrounding oedema on T2 weighted images with a margin of 2–2.5 cm. After 44–46 Gy, the treatment volume was reduced to encompass the contrast enhancing tumour on T1 weighted images plus 1.5–2 cm margins. The mean boost dose was 14–16 Gy.

TMZ was given to patients at a dose of 75 mg/ m^2^/day, 7 days per week, during the course of RT for approximately 6 weeks. After the completion of RT, patients were received an average of 6 cycles (range, 1–6) of adjuvant TMZ (150 –200 mg/m^2^/day, 5 days during each 28-day).

Forty (80%) of 50 patients were given higher or equal than 60 Gy RT, thirty nine (78%) of 50 patients received concurrent TMZ, 75 mg/m^2^ and 21 (42%) patients completed the six cycles of adjuvant TMZ. Ten (20%) of patients were given palliative RT (lower than 60 Gy), 11 patients (32%) were not given concurrent TMZ and 29 patients were not given adjuvant TMZ due to lower performance status, comorbidities, more symptoms etc. Characteristics of the patients are summarized in Table 1.

Age, sex, KPS, tumour diameter, type of surgery, dose of RT, concurrent and 6 cycles of adjuvant TMZ were analysed as a prognostic factors.

### Statistical analysis

The statistical analysis was performed by SPSS 13. version. The primary end point was the overall survival and the progression free survival (PFS). The overall survival was estimated from the date of the histopathologic diagnosis (in 5 patients at radiological diagnosis) to the date of death or last follow-up. The overall survival was analyzed by the Kaplan-Meier method. The survival curves were compared by the log rank test. Patients were categorized in some variables including patient characteristics such as age (<70 or ≥70 years), sex, KPS (<70 or ≥70), tumour diameters (<5 cm or ≥ 5 cm) treatment parameters such as surgery (gross-total or subtotal resection), concurrent and 6 cycles of adjuvant TMZ. The effect of these variables on the survival was assessed by using a multivariate Cox-regression model.

## Results

**FIGURE 1:**
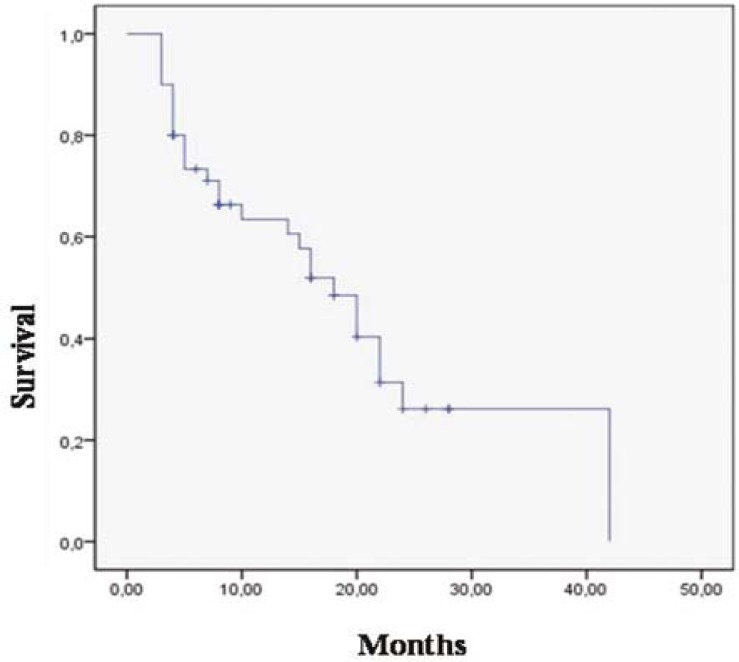
Oveall survival for 50 patients with glioblastoma multiforme.

Median follow up time was 10 (months range; 3–42 months). One-and 2-year survival rates were 46% and 20%, respectively. While 36 (72%) of 50 patients died, 14 (28%) of them are alive (Figure 1).

**FIGURE 2:**
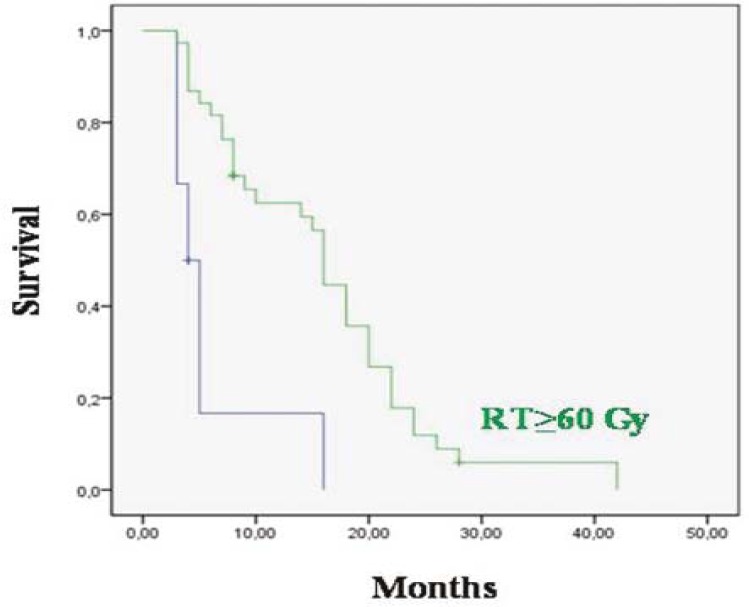
Overall survival for 40 patients who received ≥60 Gy RT and for 10 of them who received <60 Gy (p=0.0001).

**FIGURE 3:**
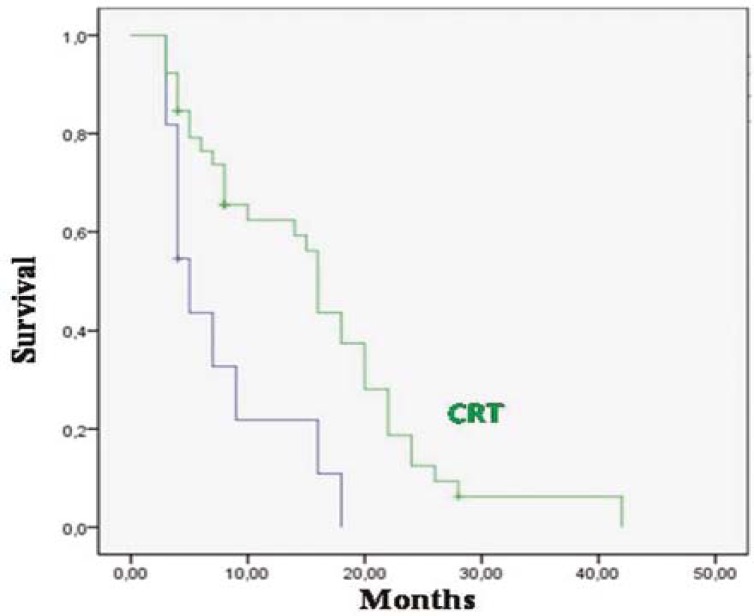
Overall survival for 39 patients who received concurrent chemo-radiotherapy.

**FIGURE 4:**
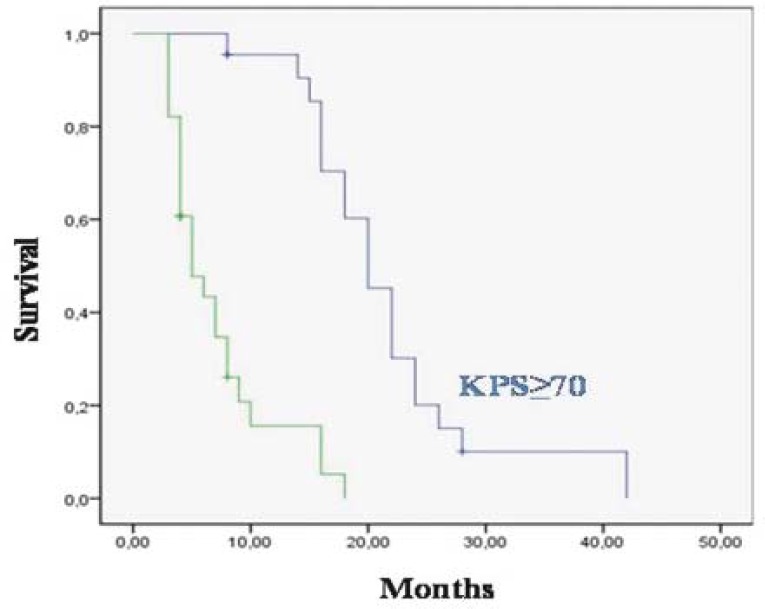
Overall survival 26 patients with Karnofsky performance status (KPS) ≥70 and 7and for 24 patients with KPS<70.

The median overall survival was 20 months for patients who received ≥60 Gy RT and 5 months for the <60 Gy (p 0.0001) (Figure 2); 18.9 months for patients who received concurrent TMZ with RT and 7.5 months for patients who did not receive it (p=0.0001) (Figure 3); 28 months for patients who have been completing 6 cycles of adjuvant TMZ and 7 months for patients who have not been completing 6 cycles of therapy (p=0.0001). The median overall survival was 27 months for patients who had KPS≥70 and 7 months who had KPS<70 (p=0.0001) (Figure 4); 17 months for patients with age <70 and 13 months with ≥70 (p=0.005); 24 months for patients who had gross total resection, 6.7 months for patients who were diagnosed with biopsy and 8.2 months for patients who were diagnosed with radiology (p=0.0001).

One- and 2-year progression free survival (PFS) rates were 40% and 16%, respectively. Median PFS was 11 months for patients completing 6 cycles of adjuvant TMZ and 5.2 months for patients not completing 6 cycles of therapy. The difference between the two groups was significant (p=0.0001). The median PFS survival was 10.7 months for patients who had KPS≥70, 5.3 months for those who had KPS<70 (p=0.0001). Median PFS was 8.7 months for patients who were given ≥60 Gy and 4.5 months who were given <60 Gy (p=0.0001). However, the comparison of PFS between the patients of age ≥70 and those of age <70 (7.9 months *vs.* 7.0 months) showed no significant difference (p=0.358).

A multivariate Cox regression model was used to test the effect of age, sex, KPS, extent of surgery, tumour dimension (<5cm *vs.* ≥5cm), full dose radiotherapy (≥60Gy *vs.* <60Gy) concurrent TMZ and adjuvant TMZ treatment (adjuvant therapy with 6 cycles of TMZ group *vs.* <6 cycles of TMZ group) on the overall survival. The prognostic factors important for the overall survival were full dose RT (≥60 Gy) (p=0.005) and the application of adjuvant TMZ for 6 cycles (p=0.009) (Table 1).

### Toxicity

The most common non-hematologic adverse effect was grade I nausea, which could be controlled by anti-emetics. It was seen in 34% of patients during the course of concurrent RT and TMZ and in 39% of patients during adjuvant TMZ. During the concomitant TMZ treatment, 2 patients (4%) experienced grade III or IV neutropenia and one patient (2%) had grade III or IV thrombocytopenia. Grade III or IV thrombocytopenia was seen in 3 patients (6%) during the adjuvant TMZ treatment. Only one patient discontinued the treatment due to hematologic toxicity. At the median follow up of 10 months we didn’t see any late toxic effect.

## Discussion

Patients with GBM, which is the most common primary brain tumour in adults, have a median overall survival rate of approximately 15 months, even with the aggressive resection, RT with concurrent and adjuvant CT.^11^ Despite modern treatment techniques, tumours virtually always recur, usually arising within <2 cm of the prior resection margin. A long term overall survival is less than 5%.^13^,^14^

A recent randomized trial by the European Organization for Research and Treatment of Cancer (EORTC) and the National Cancer Institute of Canada (NCIC) demonstrated that concomitant radiotherapy plus continuous daily TMZ followed by adjuvant TMZ significantly prolonged the survival in patients with glioblastoma. Five hundred seventy-three patients were randomly assigned to either standard RT alone or RT and concomitant and maintenance administration of TMZ (TMZ/ RT). RT consisted of 30 fractions of 2 Gy each, administered Monday–Friday for 6–7 weeks. TMZ chemotherapy, at a low dose of 75 mg/m^2^, was administered daily, including weekends, from the first to the last day of RT, for up 49 days. After a 4-week break, patients were to receive up to six cycles of maintenance TMZ (150–200 mg/m^2^) daily for 5 days every 4 weeks. The median survival was 14.6 months for the radiotherapy plus TMZ group and 12.1 months for the radiotherapy alone group (P<0.001). PFS improved significantly in the radiotherapy plus TMZ group compared to the radiotherapy group (7.2 *vs.* 5 months, P<0.001). The 2-year survival rate was 26.5 for the combined treatment group.^11^

After the report of the EORTC-NCIC regimen of the maximal surgical resection followed by concurrent RT and TMZ followed by at least 6 monthly adjuvant TMZ cycles became the standard of care for newly diagnosed GBM. Patients in the EORTC-NCIC phase 3 study without disease progression discontinued adjuvant TMZ after six monthly cycles in part because of concerns regarding long-term toxicity such as myelodysplasia. Although no data are yet available demonstrating the improvement in the survival with the prolonged adjuvant therapy, reports of serious side effects from prolonged TMZ use are rare^15^, and most neuro-oncologists in the United States advocate at least 12 post-RT adjuvant TMZ cycles for patients without disease progression. Ongoing clinical trials also typically incorporate 12 adjuvant TMZ cycles.^16^

In Erpolat *et al.* study, all patients received concomitant TMZ and radiotherapy at a daily dose of 75 mg/m^2^, with or without adjuvant TMZ as recommended by the EORTC/NCIC trial. The overall survival and PFS were analyzed based on the comparison of the two groups whether or not they received adjuvant TMZ. Although the overall survival significantly increased in the adjuvant TMZ group (18.9 months *vs.* 9.8 months, P=0.015), the log rank test did not show significance for PFS (P=0.108). On both univariate and multivariate analysis, it was shown that the application of at least 4 cycles of adjuvant TMZ improved the overall survival and PFS. Their results suggest that at least 4 cycles of adjuvant TMZ should be added to the concomitant therapy to ensure the sufficient exposure to the drug.^17^

We have also shown that the treatment with concurrent and adjuvant TMZ is associated with a significant increase in the overall survival compared to without concurrent and/or adjuvant TMZ alone on univariate analyses. The median overall survival was 18.9 months for patients who received concurrent TMZ with RT and 7.5 months for patients not receiving concurrent TMZ (log-rank, p=0.0001) and 28 months for patients who completing 6 cycles of adjuvant TMZ and 7 months for patients not completing 6 cycles of therapy (log-rank, p=0.0001).

Hegi *et al.* investigated the relationship between promoter methylation of the MGMT DNA-repair gene and responsiveness to TMZ.^18^ The authors demonstrated that the survival benefit of TMZ was limited to those patients with tumour containing methylated MGMT promoters. Among these patients, the median survival with radiotherapy and TMZ was 21.7 months (95% CI 17.4–30.4), compared to 15.3 months with radiotherapy alone (95% CI 13.0–20.9; p=0.007). An insignificant survival difference was demonstrated between treatment groups in patients with unmethylated MGMT promoters. This finding points to a genetic basis for the efficacy of TMZ and underscores the role of gene expression in optimal patient selection.^3^,^19^ Although these articles represent a substantial step forward in the treatment of GBM, at this point it is too early in standard clinical practice to choose the TMZ treatment according to molecular criteria alone.^20^

Curran *et al.*^12^ reported that the prognosis of malignant gliomas is relevant to the clinicopathologic variables such as treatment. Recursive partitioning analyses of the prognostic factors identified the five major variables including age, tumour type, performance status, mental status and treatment (extent of surgery and radiation dose). According to this classification, compared to young patients with good performance-mental status, the expectancy for those who have poor performance-mental status and/or advanced ages is dismal. For this reason, the prediction of the treatment response is related to this classification.

In our study, multivariate and univariate analyses were performed based on these variables and demonstrated that the most important prognostic factors were: 6 cycles of adjuvant TMZ application, full dose radiotherapy (≥60Gy *vs.* <60Gy) and extent of surgery (gross total *vs.* others) for only the overall survival. However, younger age (<70), concurrent TMZ and good KPS (≥70) were significant for the overall survival on the univariate analysis but it did not reach a significant value on the multivariate analysis.

Advanced age has been associated not only with a poor prognosis but also with a reduced tolerance of the treatment and a decreased efficacy of the therapy.^12^ The role of RT combined with concomitant and adjuvant TMZ, as established by the EORTC/NCIC trial as the standard treatment for patients with newly diagnosed GBM, remains unclear for the group of elderly patients. Gerstein *et al.* very recently reported that RT with concomitant TMZ is a feasible regimen with acceptable toxicity in elderly patients.^21^ The promising outcome in patients with a good performance status and patients with gross total resections are notable. In our study, median overall survival was 17 months who age <70 and 13 months who age ≥70 (p=0.005).

The radiation therapy remains the most effective adjuvant modality in the management of GBM and the overall survival time appears to be correlated with the total dose delivered.^19^,^20^ In our results on univariate and multivariate analysis ≥60 Gy RT was significantly improved for the overall survival (20 months *vs.* 5 months, p=0.0001 and p=0.005, respectively).

Mirimanoff *et al.* evaluated in EORTC/NCIC trial’s patients whether the recursive partitioning analysis (RPA) retains its overall prognostic value and what the benefit of the combined modality is in each RPA class.^24^ The overall survival was statistically different among RPA classes III, IV, and V, with median survival times of 17, 15, and 10 months, respectively, and 2-year survival rates of 32%, 19%, and 11%, respectively (p*<*0.0001). The survival with combined TMZ/RT was higher in RPA class III, with 21 months median survival time and a 43% 2-year survival rate, *vs.* 15 months and 20% for RT alone (p=0.006). In RPA class IV, the survival advantage remained significant, with median survival times of 16 *vs.* 13 months, respectively, and 2-year survival rates of 28% *vs.* 11%, respectively (p*=*0.0001). In RPA class V, however, the survival advantage of RT/TMZ was of borderline significance (p*=*0.054).

Our results demonstrated that a good performance status (KPS≥70) was significant for the overall survival on univariate analysis but it did not reach a significant value on the multivariate analysis.

Our study has several shortcomings. Firstly, it is retrospective in nature. Secondly, it has small sample size. However, the survival for patients who received TMZ according to the Stupp protocol was well, with a 1 and 2-year overall survival of 46% and 20% respectively. In addition, traditional prognostic factors were confirmed in our results, as age (<70), KPS (≥70), radiotherapy dose (≥60Gy), and gross total resection status were all favourable characteristics.

The results of our study confirm the efficiency of radiotherapy plus concomitant and adjuvant TMZ, with an acceptable toxicity in patients. We suggest that at least 6 cycles of adjuvant TMZ should be administered to obtain a benefit from the adjuvant treatment. Prospective randomized trials include the combination of TMZ with other drugs which need to be tested with large series of patients.

## Figures and Tables

**TABLE 1:** Demographic characteristics of the patients

**Characteristics**	**Number of patients**	**%**	**Median survival (Months)**	**12-month survival %**	**24-month survival %**	**Univariate analysis p**	**Multivatiate analysis p**
**Sex**						0.279	0.759
Female	16	32	12	38.2	0		
Male	34	68	18	50.1	23.5	0.279	0.759
**Age (yr)**	**0.005**						0.601
< 70	14	28	17	41.7	21.1	**0.005**	0.601
≥ 70	36	72	13	35.7	0		
**KPS**	**0.0001**						0.151
< 70	24	48	7	0	0		
≥ 70	26	52	27	76.7	35.2	**0.0001**	0.151
**Tumour diameter (cm)**	0.849						0.928
< 5	15	30	17	61.5	38.5	0.849	0.929
≥ 5	35	70	15	40	30		
**Extent of surgery**						**0.0001**	**0.0001**
Total	22	44	24	95.5	57.3	**0.0001**	**0.0001**
Subtotal	9	18	6.7	11	0		
Biopsy	14	28	7.2	7	0		
Radiological	5	10	8.2	20	0		
**≥60 Gy RT**	**0.0001**						**0.005**
Yes	40	80	20	49.9	22.9	**0.0001**	**0.005**
No	10	20	5	0	0		
**Concurrent CT-RT**	**0.005**						
Yes	39	78	18.9	48.6	24.3	**0.005**	
No	11	22	7.5	9.1	0		
**Adjuvant CT (6 cycles)**	**0.0001**						**0.009**
Yes	21	42	28	80.7	37.8	**0.0001**	**0.009**
No	29	58	7	6.9	0		

KPS = Karnofsky performance status; RT = radiotherapy; CT = chemotherapy
